# Ubiquinone Analogs: A Mitochondrial Permeability Transition Pore-Dependent Pathway to Selective Cell Death

**DOI:** 10.1371/journal.pone.0011792

**Published:** 2010-07-26

**Authors:** Flavien Devun, Ludivine Walter, Julie Belliere, Cécile Cottet-Rousselle, Xavier Leverve, Eric Fontaine

**Affiliations:** 1 INSERM, U884, F-38041, Grenoble, France; 2 Université Joseph Fourier, Laboratoire de Bioénergétique Fondamentale et Appliquée, F-38041, Grenoble, France; University of Florida, United States of America

## Abstract

**Background:**

Prolonged opening of the mitochondrial permeability transition pore (PTP) leads to cell death. Various ubiquinone analogs have been shown to regulate PTP opening but the outcome of PTP regulation by ubiquinone analogs on cell fate has not been studied yet.

**Methodology/Principal Findings:**

The effects of ubiquinone 0 (Ub_0_), ubiquinone 5 (Ub_5_), ubiquinone 10 (Ub_10_) and decyl-ubiquinone (DUb) were studied in freshly isolated rat hepatocytes, cultured rat liver Clone-9 cells and cancerous rat liver MH1C1 cells. PTP regulation by ubiquinones differed significantly in permeabilized Clone-9 and MH1C1 cells from that previously reported in liver mitochondria. Ub_0_ inhibited PTP opening in isolated hepatocytes and Clone-9 cells, whereas it induced PTP opening in MH1C1 cells. Ub_5_ did not affect PTP opening in isolated hepatocytes and MH1C1 cells, but it induced PTP opening in Clone-9 cells. Ub_10_ regulated PTP in isolated hepatocytes, whereas it did not affect PTP opening in Clone-9 and MH1C1 cells. Only DUb displayed the same effect on PTP regulation in the three hepatocyte lines tested. Despite such modifications in PTP regulation, competition between ubiquinones still occurred in Clone-9 and MH1C1 cells. As expected, Ub_5_ induced a PTP-dependent cell death in Clone-9, while it did not affect MH1C1 cell viability. Ub_0_ induced a PTP-dependent cell death in MH1C1 cells, but was also slightly cytotoxic in Clone-9 by an oxidative stress-dependent mechanism.

**Conclusions/Significance:**

We found that various ubiquinone analogs regulate PTP in different ways depending on the cell studied. We took advantage of this unique property to develop a PTP opening-targeted strategy that leads to cell death specifically in cells where the ubiquinone analog used induces PTP opening, while sparing the cells in which it does not induce PTP opening.

## Introduction

Mitochondria are involved in several physiological processes including energy metabolism, calcium homeostasis and programmed cell death [1], [2], [3]. Numerous mitochondrial proteins, which have no pro-apoptotic activity when they remain inside mitochondria, promote cell death once released into the cytosol [4]. Both extra-mitochondrial and intra-mitochondrial signaling pathways can trigger the release of the mitochondrial pro-apoptotic proteins [2].

The mitochondrial permeability transition consists of a sudden non-specific increase in the permeability of the inner membrane [5], [6]. A prolonged mitochondrial permeability transition results in a drastic ATP synthesis inhibition through the collapse of the proton-motive force, a dramatic increase in ROS production and the release of the mitochondrial pro-apoptotic proteins [7], [8], [9]. Permeability transition is due to the opening of an inner membrane channel [10]: the Permeability Transition Pore (PTP).

Matrix Ca^2+^ is the single most important factor for PTP opening. The amount of matrix Ca^2+^ required to open the pore is modulated by a number of factors. The “PTP-inhibitors” and the other so-called “PTP-inducers” designate factors that increase and decrease the amount of Ca^2+^ required to induce PTP opening [6]. Cyclosporin A (CsA) is the reference PTP inhibitor. It inhibits PTP opening by detaching Cyclophilin D (CyP-D) from the other components of the pore [11].

In primary and cultured cells, several drugs known to inhibit PTP opening also decrease cell death in response to various cytotoxic insults [12], [13], [14]. In animal models, the inhibition of PTP opening by either CsA or genetic ablation of CyP-D provides strong protection from reperfusion injury [15], [16], [17]. In humans, the first clinical trial has recently shown that CsA treatment reduces infarct size after reperfusion of a coronary thrombosis [18]. These data suggest that PTP inhibition can be beneficial in particular pathological conditions, most likely through its effect on cell death. On the other hand, resistance to Doxorubicin has been shown to be related to PTP inhibition in a human chronic myelogenous leukemia cell line [19], while hepatocarcinogenesis with 2-acetylaminofluorene is preceded by PTP inhibition [20]. Thus, PTP inhibition might in some cases hamper cancer treatments or eventually participate in carcinogenesis.

Because prolonged PTP opening leads to cell death, the PTP represents a cellular target for the commitment to cell death [21]. Indeed, pharmacological agents used in anti-cancer therapy have been reported to target the PTP and to induce cell death via PTP opening [1], [21]. Ideally, drugs used for the treatment of malignancies would be far more toxic for cancer cells than for normal cells. However, a PTP-targeted drug able to selectively open the PTP in cancerous cells only remains to be developed.

In a series of experiments conducted with isolated rat liver mitochondria, we have shown that several ubiquinone analogs regulate PTP opening [22], [23], [24], [25]. Three functional classes of quinones were defined, the PTP-inhibitory quinones, the PTP-inducing quinones and the PTP-inactive quinones that counteract the effects of both inhibitory and inducing quinones [24], [25]. To date, few studies have reported a preventive effect of ubiquinone analogs in a model of cell death. DUb, a PTP-inhibitor quinone in the liver, has been shown to prevent PTP opening-induced cell death in HL 60 cells [26]. In contrast, although Ub_0_ is more potent than CsA at PTP inhibition in liver and skeletal muscle mitochondria [27], Ub_0_ was ineffective in preventing PTP opening-induced cell death in HL 60 cells [26].

To clarify this issue, and because PTP regulation can vary depending on the tissue studied [27], we have begun a comprehensive study of PTP regulation in different cell lines. This work presents the effect of four ubiquinone analogs on three different rat liver cell lines. We confirm that ubiquinone analogs regulate PTP opening in the different cells tested. However, we found that a number of ubiquinone analogs may regulate PTP in different ways depending on the cell studied. We took advantage of this unique property to develop a PTP opening-targeted strategy that leads to cell death specifically in cells where the ubiquinone analog used induces PTP opening.

## Materials and Methods

### Cells

Clone-9 and MH1C1 cells are, respectively, non-cancerous and cancerous rat hepatocyte cell lines. Clone-9 and MH1C1 cells were maintained in exponential growth phase using Dulbecco's modified Eagle's medium supplemented with 10% fetal bovine serum for Clone-9, and Ham's F12K medium supplemented with 2.5% fetal bovine serum and 15% horse serum for MH1C1. Both media were supplemented with 2 mM glutamine, 1mM sodium pyruvate, 1% non-essential amino acids, 50 units/ml penicillin, and 50 µg/ml streptomycin. Hepatocytes were isolated according to Berry and Friend's methodology, modified by Groen et al [28].

### Ca^2+^ retention capacity

Non confluent cultured cells, harvested by trypsinization, washed with PBS or freshly isolated hepatocytes were permeabilized immediately before use by incubation of 5×10^6^ cells under agitation for 2 min at 25°C in a Ca^2+^ free medium (Chelex resin, overnight, 4°C) containing 250 mM sucrose, 1 mM Pi-Tris, 10 mM Tris-MOPS (pH 7.4) and 50 µg/ml digitonin. Measurements of Ca^2+^ were performed fluorimetrically at 25°C with a PTI Quantamaster C61 spectrofluorometer equipped with magnetic stirring and thermostatic controls. Extra-mitochondrial Ca^2+^ was measured in the presence of 1 µM Calcium Green-5N with excitation and emission wavelengths set at 506 and 530 nm, respectively. The Ca^2+^ uptake and Ca^2+^ release of digitonin permeabilized cells were measured by loading cells with trains of Ca^2+^ pulses at constant time intervals.

### ROS production

Cells were incubated in a medium containing 250 mM sucrose, 1 mM Pi-Tris, 10 mM Tris-MOPS, and 5 µM H_2_DCFDA. Measurement of H_2_DCFDA oxidation were performed fluorimetrically at 37°C (with excitation and emission wavelengths set at 506 and 521 nm, respectively) with a PTI Quantamaster C61 spectrofluorometer equipped with magnetic stirring and thermostatic controls.

### Cells Staining

Clone-9 cells were labeled with the lipophilic dye PKH26 according to the manufacturer's instructions. This non-toxic fluorescent dye binds irreversibly to the cell membrane without affecting cell growth. Therefore, upon cell division, the probe is partitioned equally between each daughter cell, but does not transfer to co-cultured cells [29].

### Cells treatment and cell death analysis

Cells were exposed to the indicated concentrations of ubiquinone analogs for 30 min at 37°C in a serum-free medium. Cells were then harvested and incubated in a complete medium at 37°C for 24 h. Annexin V-positive cells were quantified by flow cytometry using a FACSCan flow cytometer (Becton-Dickinson). Cells (1×10^6^/ml) were exposed to 5% v/v annexin V-FluoProbes Alexa 488 for 15 min at room temperature. For each sample, a minimum of 10,000 events were analyzed.

### Reagents

Ub_0_ (2,3-dimethoxy-5-methyl-1,4-benzoquinone or coenzyme Q_0_), Ub_5_ (2,3-Dimethoxy-*5*-methyl-6-(3-methyl-2-butenyl)-1,4-benzoquinone or coenzyme Q_1_), Ub_10_ (2,3-Dimethoxy-5-methyl-6-geranyl-1,4-benzoquinone or coenzyme Q_2_), DUb (2,3-Dimethoxy-5-methyl-6-decyl-1,4-benzoquinone) and CsA were purchased from Sigma. Calcium Green-5N, H_2_DCFDA and Annexin V-FluoProbes Alexa 488 were purchased from Molecular Probes. Ham's F12K medium was purchased from Gibco, Fetal Bovine Serum from Biotech and trypsin from Jacques Boy. The remaining reagents were purchased from Sigma.

### Statistics

Stastistical analyses were performed using two-tailed unpaired Student's t tests with equal variances.

## Results

The Ca^2+^ retention capacity (CRC) represents the minimum Ca^2+^ load required to induce PTP opening in an entire population of mitochondria. Therefore, CRC measurement represents a suitable method to quantify and compare the potency of different PTP regulators. The CRC is measured by loading mitochondria with train of Ca^2+^ pulses until a rapid Ca^2+^ release occurs. This event is accompanied by mitochondrial swelling and membrane depolarization, and is prevented by CsA [27].

The CRC measurement can be performed equally well in isolated or *in situ* mitochondria (i.e. in digitonin permeabilized cells). However, because PTP regulation may be very sensitive to the conditions of incubation used [27], [30], we first checked whether PTP regulation by ubiquinone analogs in permeabilized rat hepatocytes was identical to that previously measured in isolated rat liver mitochondria.

**Figure 1:**
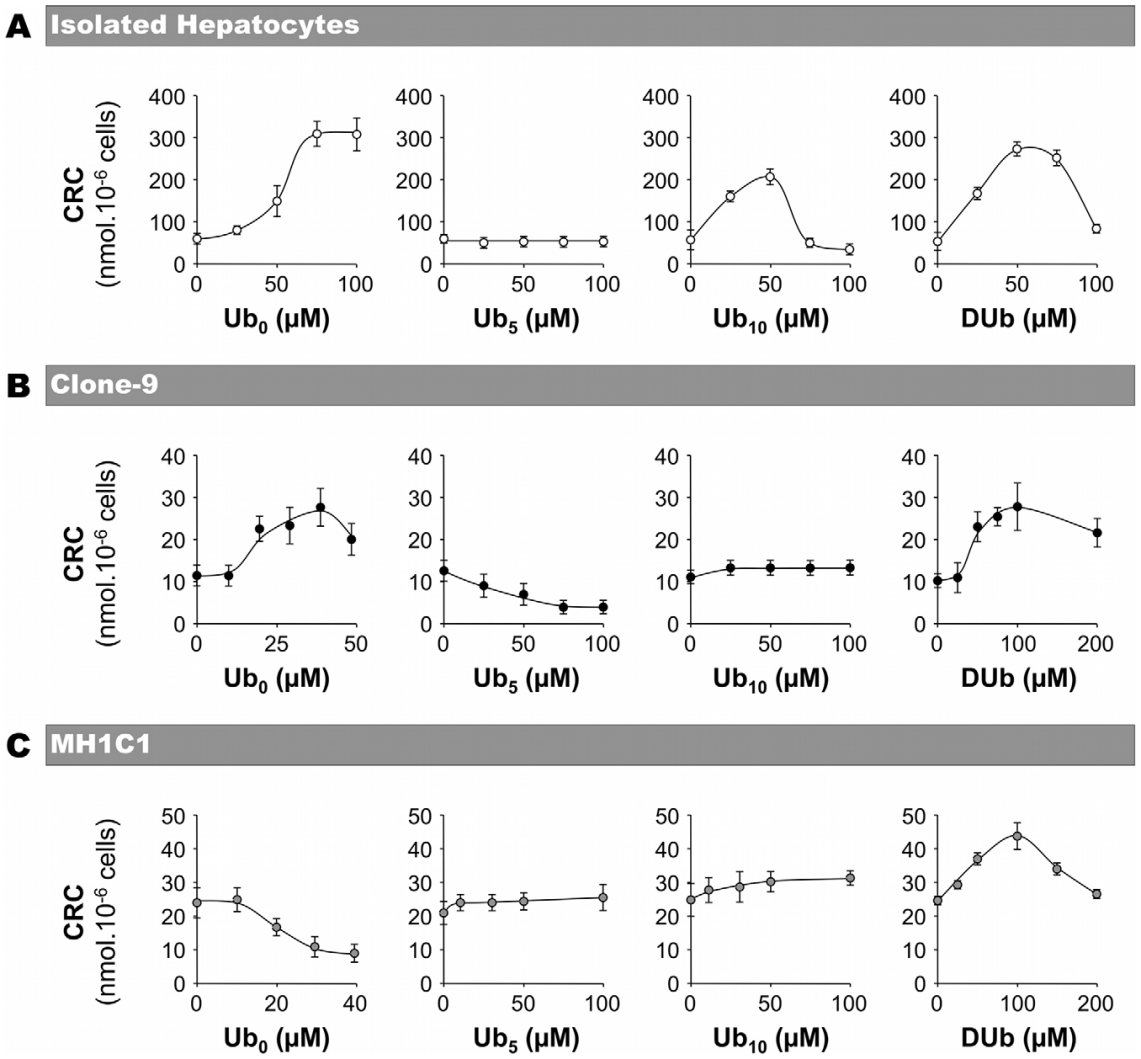
The regulation of PTP opening by ubiquinone analogs depends on the cell lines. The incubation medium contained 250 mM sucrose, 1 mM Pi-Tris, 10 mM Tris-MOPS, 5 mM succinate-Tris, 50 µM digitonin and 1 µM Calcium Green-5N. The final volume was 2 ml, pH 7.4, 25°C. Experiments were begun by the addition of 5.10^6^ cells (isolated rat hepatocytes, MH1C1 or Clone-9) followed by the addition of ubiquinone 0 (Ub_0_), ubiquinone 5 (Ub_5_), ubiquinone 10 (Ub_10_) or decyl-ubiquinone (DUb) at the indicated concentrations. After 2 min of incubation, the Ca^2+^ Retention Capacity (CRC) was measured by adding Ca^2+^ pulses every 90 s until PTP opening. Results are mean ± S.D. of at least four independent experiments.

As shown in Figure 1A, Ub_0_ and DUb increased the CRC (i.e. inhibited PTP opening) in rat hepatocytes in a concentration-dependent manner up to an optimal concentration beyond which Ub_0_ and DUb became less potent at PTP inhibition. Ub_10_ inhibited PTP opening at low concentrations, but activated PTP opening at high concentration in rat hepatocytes. Ub_5_ did not affect PTP regulation in rat hepatocytes. These results are in total agreement with those previously found with isolated rat liver mitochondria [23], [24], [25], indicating that the model used (i.e. isolated mitochondria or *in situ* mitochondria) did not influence the effect of the tested ubiquinone analogs at PTP regulation.

We next studied the effects of Ub_0_, Ub_5_, Ub_10_ and DUb on the CRC of two other digitonin permeabilized rat liver cells, namely cultured rat liver Clone-9 cells and cancerous rat liver MH1C1 cells. As shown in Figure 1, PTP opening in the absence of ubiquinone analogs occurred at approximately 60, 10 and 25 nmol Ca^2+^ per million cells in isolated hepatocytes, Clone-9 and MH1C1, respectively. Such differences in the basal CRC are expected [31] and may be at least partly related to differences in the number of mitochondria per cell. Note, however, that CsA inhibited PTP opening in the three different cell lines (data not shown).

In immortalized Clone-9 cells (Figure 1B), Ub_0_ and DUb were PTP-inhibitors as observed in permeabilized hepatocytes, although the optimal concentration varied with the cell line studied. Surprisingly, contrary to what occurred in permeabilized hepatocytes, Ub_5_ favored PTP opening, whereas Ub_10_ was ineffective in Clone-9 cells. In cancerous rat liver MH1C1 cells (Figure 1C), Ub_0_ favored PTP opening, Ub_5_ and Ub_10_ were ineffective, whereas DUb inhibited PTP opening. These data indicate that the effects of ubiquinone analogs on PTP regulation dramatically differed according to the type of cell used.

**Figure 2:**
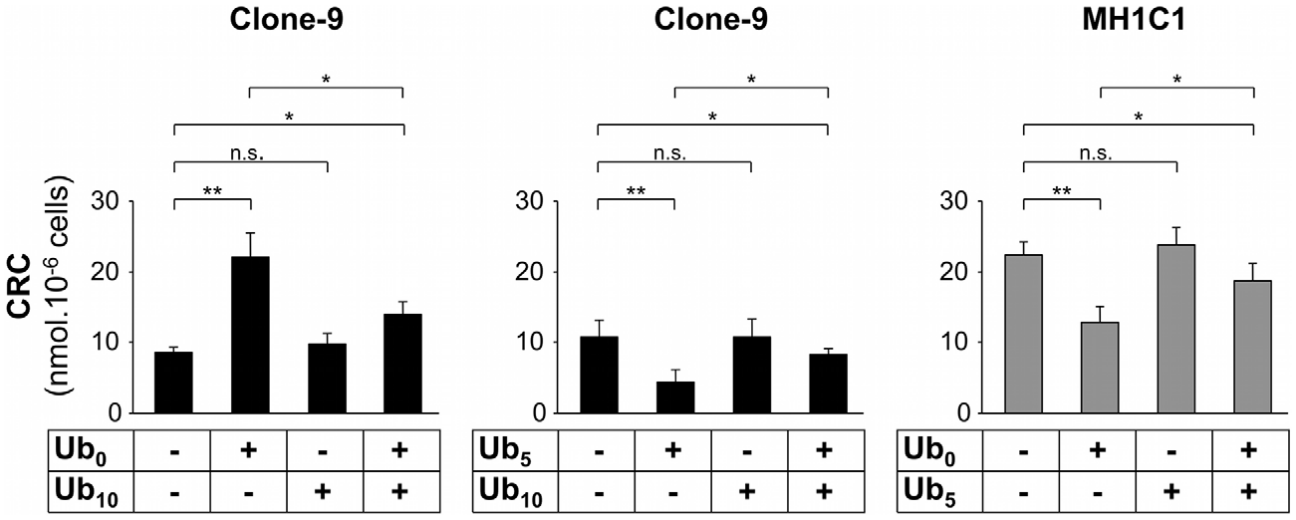
Competition between PTP-inactive and PTP-active ubiquinone analogs on PTP regulation in Clone-9 and MH1C1 cells. The Ca^2+^ Retention Capacity was measured as in Fig. 1. When indicated, 20 µM Ub_0_, 100 µM Ub_5_ or 100 µM Ub_10_ were added. The results represent the means ± S.D. of three independent experiments. *, p≤0.05; **, p≤0.01, unpaired Student's t test.

In isolated rat liver mitochondria, we have shown that PTP-inactive quinones were able to counteract the effects of both inhibitory and inducing quinones, suggesting the existence of a common site of action for which the ubiquinone analogs compete [25]. In order to check whether these competitions persist in cells in which PTP regulation differs from that observed in liver mitochondria, permeabilized Clone-9 and MH1C1 cells were exposed to a combination of PTP-active plus PTP-inactive quinones. Results are presented in Figure 2. In Clone-9, the PTP-inactive quinone Ub_10_ was able to counteract the effect of the PTP- inhibitory Ub_0_ and of the PTP-inducing Ub_5_. Similarly, in MH1C1, the PTP-inactive quinone Ub_5_ was able to counteract the inducing effect of Ub_0_. Therefore, despite the fact that PTP regulation by quinone changes according to the cell studied, this data suggests that the competition between 3 functionally classes of quinones is a ubiquitous phenomenon.

Because PTP opening is expected to induce cell death, we next verified whether the changes observed in PTP regulation were associated with consecutive changes in cell death regulation. In other words, we hypothesized that ubiquinone analogs were capable of inducing cell death selectively in cells in which they favored PTP opening.

**Figure 3:**
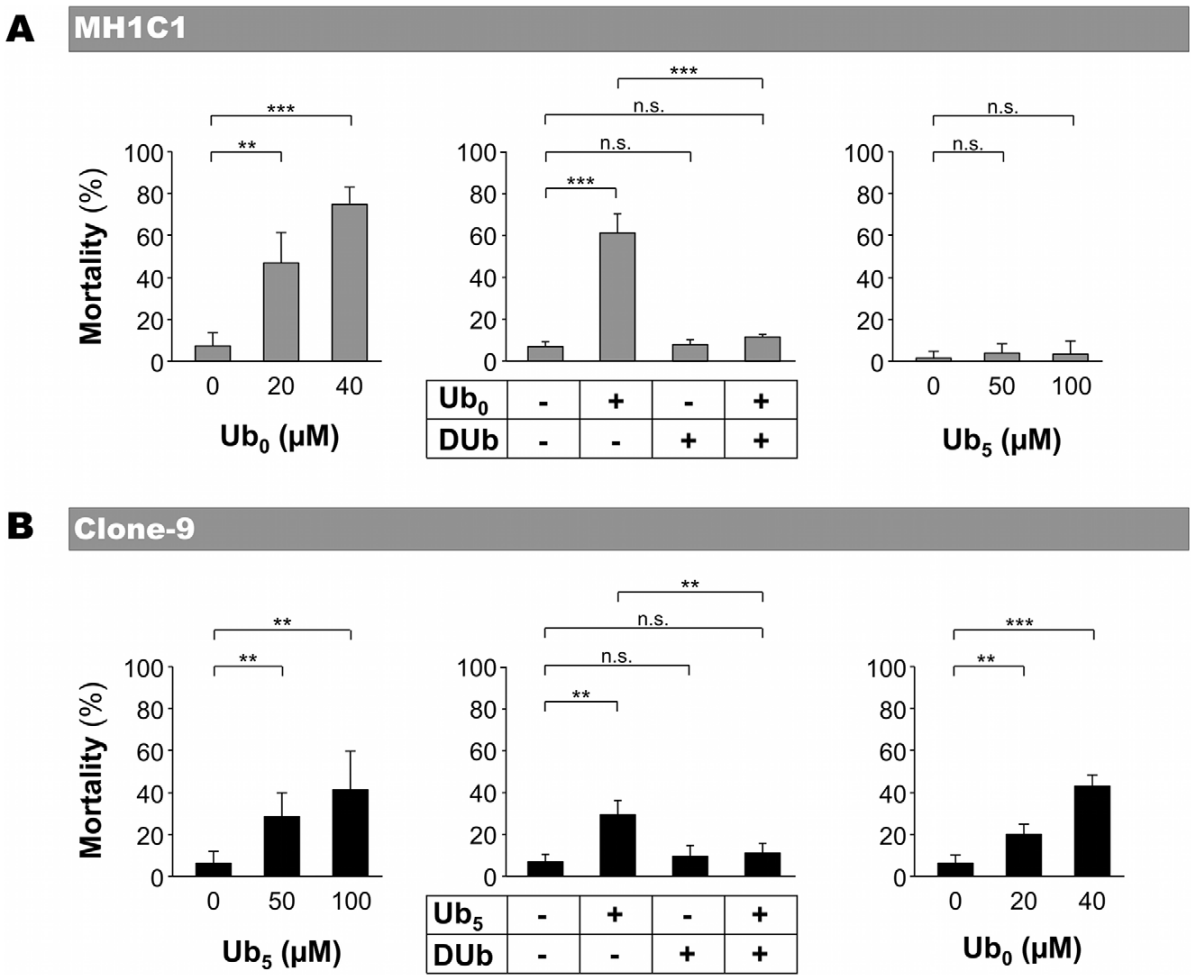
Effect of ubiquinone analogs on MH1C1 and Clone-9 viability. MH1C1 and Clone-9 cells were exposed for 30 min to serum-free culture medium supplemented or not with the indicated concentrations of Ub_0_ or Ub_5_ (left and right panels). When used in combination (middle panels), the concentrations were 20 µM for Ub_0_, 50 µM Ub_5_ and 100 µM for DUb. Cells were then incubated in normal medium for 24 h. The percentage of mortality represents the proportion of Annexin V-positive cells measured by flow cytometry. The results represent the means ± S.D of at least four independent experiments. **, p≤0.01; ***, p≤0.001, unpaired Student's t test. In preliminary experiments, we found that Annexin V-positive cells were mostly Propidium iodide positive.

In MH1C1 cells (Figure 3A), PTP-inducer Ub_0_ induced cell death in a concentration-dependent manner. Moreover, Ub_0_-induced cell death was prevented by PTP-inhibitor DUb (Figure 3A) and by CsA (not shown), confirming that Ub_0_-induced cell death was due to PTP opening in that cell line. As expected, PTP-inactive Ub_5_ and PTP-inhibitor DUb did not induce any significant toxicity.

In Clone-9 cells (Figure 3B), PTP-inducer Ub_5_ induced cell death in a concentration-dependent manner. As expected, Ub_5_-induced cell death was prevented by PTP-inhibitors DUb (Figure 3B) and by CsA (not shown). Surprisingly, Ub_0_ and DUb, which both inhibited PTP opening in that cell line (see Figure 1), affected cell viability in a different manner. DUb did not induce any significant toxicity, whereas Ub_0_ induced Clone-9 cell death.

**Figure 4:**
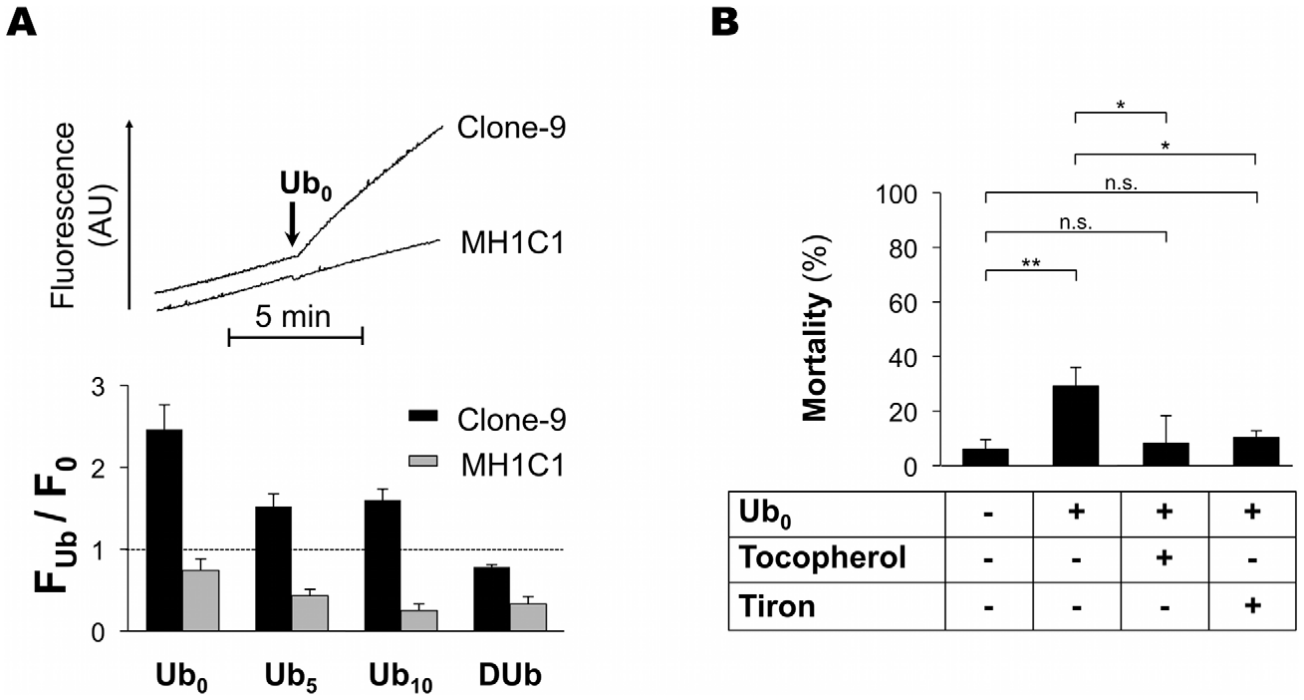
The effect of ubiquinone analogs on ROS production depends on the cell lines. **A** - The incubation medium contained 250 mM sucrose, 1 mM Pi-Tris, 10 mM Tris-MOPS, and 5 µM H_2_DCFDA. The final volume was 2 ml, pH 7.4, 37°C. Experiments were begun by the addition of 5.10^6^ cells (MH1C1 or Clone-9) followed by the addition of 20µM Ub_0_, 100µM Ub_5_, 100µM Ub_10_ or 100µM DUb. F_Ub_/F_0_ represents the change in fluorescence during 5 min after the addition of ubiquinone analog divided by the change in fluorescence during 5 min before the addition of ubiquinone analog. Results are mean ± S.D. of at least three independent experiments. **B** - Clone-9 cells were exposed for 30 min to serum-free culture media supplemented or not with 20 µM Ub_0_, 200 µM tocopherol or 1 mM tiron. Cells were then incubated in normal medium for 24 h. The percentage of mortality represents the proportion of Annexin V-positive cells measured by flow cytometry. The results represent the means ± S.D of at least three independent experiments. *, p≤0.05; **, p≤0.01, unpaired Student's t test.

Ubiquinone analogs have been reported to either reduce or increase reactive oxygen species (ROS) formation [25], [26]. In order to check whether Ub_0_-induced cell death in Clone-9 was related to oxidative stress, we next measured H_2_DCFDA oxidation (i.e., ROS production) in Clone-9 and MH1C1 cells before and after the addition of Ub_0_, Ub_5_, Ub_10_ or DUb. As shown in Figure 4A, Ub_0_ dramatically increased ROS production in Clone-9, whereas it slightly decreased ROS production in MH1C1 cells. Ub_5_ and Ub_10_ also acted as pro-oxidants in Clone-9 (although they were less potent than Ub_0_), whereas they acted as antioxidants in MH1C1. Only DUb was antioxidant in the two cell lines. Confirming that Ub_0_ toxicity in Clone-9 was due to oxidative stress, tocopherol or tiron hampered Ub_0_-induced cell death in Clone-9 (Figure 4B).

**Figure 5:**
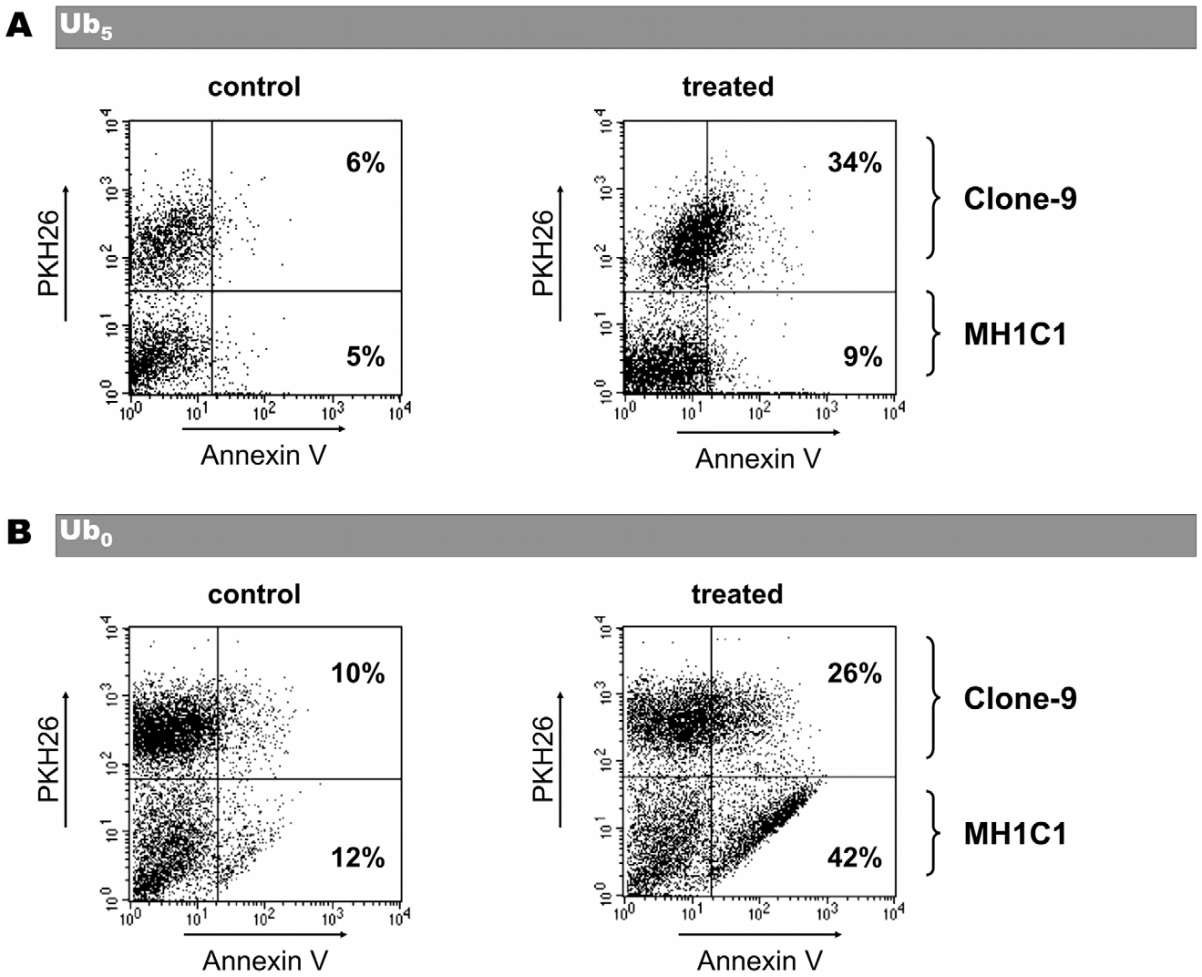
Effect of Ub_0_ or Ub_5_ on coculture of MH1C1 plus Clone-9 cells. Clone-9 cells labeled with PKH26 lipophilic dye were cocultured with MH1C1 cells for 24 h in complete F12K medium. Cocultures were exposed for 30 min to serum-free culture medium supplemented or not with 20 µM Ub_0_ or 100 µM Ub_5_. Cocultures were then incubated in complete F12K medium for 24 h. Annexin V-positive cells and PKH-26-positive cells were measured by flow cytometry. The indicated percentages represent the percentage of Annexin V-positive cells within each population in the shown experiment. Results are representative of six independent experiments.

Because Ub_5_ induced cell death in Clone-9 but not in MH1C1, we next measured the toxicity of Ub_5_ in co-cultures of MH1C1 plus Clone-9 cells. In order to distinguish the two populations of cells, Clone-9 were labeled with the fluorescent lipophilic dye PKH-26 before being co-cultured with MH1C1 in a complete F12K medium. In preliminary experiments, we first checked that PKH-26 did not affected PTP regulation or cell viability (data not shown). As shown in Figure 5A, Ub_5_ induced cell death in the Clone-9 population, but it spared the MH1C1 population.

Because a same concentration of Ub_0_ was more cytotoxic in cells in which it induced PTP opening (i.e. MH1C1) than in cells where it inhibited PTP opening (i.e. Clone-9), we also measured the toxicity of Ub_0_ in co-cultures of MH1C1 plus Clone-9 cells. As shown in Figure 5B, Ub_0_ induced cell death in the two populations but with a higher percentage of cell death in MH1C1 cells.

## Discussion

In this work we have shown that several ubiquinone analogs are able to regulate PTP opening according to the cell type. Therefore, an important and practical conclusion of this work is that PTP regulation by ubiquinones is an unpredictable phenomenon that cannot be extrapolated from results observed with isolated liver mitochondria. However, this remarkable property led us to develop a PTP-oriented strategy that allows a selective death in cells in which the ubiquinone induces PTP opening, while sparing totally (Ub_5_ in MH1C1) or partially (Ub_0_ in Clone-9) the cells in which the ubiquinone does not induce PTP opening. If every PTP inhibiting ubiquinone did not necessarily prevent cell death, all the PTP inducing ubiquinones induced cell death, confirming that PTP opening triggers the cell suicide program.

Another important finding of this work is that ubiquinone analogs are able to modulate ROS production in different way according to the cell type. Note however that within a given cell line, the way a ubiquinone analog modulates ROS production does not correlate with the way it regulates PTP opening (compare Figures 1 and 4). Indeed, ubiquinone analogs can (i) inhibit PTP opening and stimulate ROS production (Ub_0_ in Clone-9), (ii) favor PTP opening and inhibit ROS production (Ub_0_ in MH1C1 cells), (iii) favor both PTP opening and ROS production (Ub_5_ in Clone-9), (iv) inhibit both PTP opening and ROS production (DUb in Clone-9 and MH1C1 cells), (v) modify ROS production without obvious effect on PTP regulation (Ub_5_ and Ub_10_ in MH1C1 cells, Ub_10_ in Clone-9). Therefore, the effects of the ubiquinone analogs on ROS production cannot account for the effects on PTP opening, and *vice versa*.

Despite the fact that Ub_0_ is the most potent PTP inhibitor discovered so far in rat liver mitochondria [27], this work shows that Ub_0_ favors PTP opening in MH1C1 cells or induces oxidative stress in Clone-9 cells. It has been reported that Ub_0_ was ineffective in preventing cell death in HL 60 cells [26]. This may be easily explained hypothesizing that Ub_0_ may not inhibit PTP opening or may induce oxidative stress in HL 60 cells.

Some tissue-specificities in PTP regulation have been previously reported. For example, CsA does not inhibit PTP opening in a particular line of K562 cell resistant to doxorubicin [19]. The inhibition of respiratory chain complex 1 inhibits PTP opening in U937, KB and HMEC cells [31], [32], [33], whereas it does not have this effect in rat liver or rat heart mitochondria [6]. However, to the best of our knowledge, this is the first piece of evidence showing that a drug can regulate PTP opening in contradictory ways which are dependent on the cell line.

In previous works conducted with isolated rat liver mitochondria, we proposed that ubiquinone analogs might regulate PTP via a common site. This hypothesis was sustained by the fact that inactive quinones were able to counteract the effects of both inhibitory and inducing quinones. At that time, the biphasic effect of some quinones (inhibitory at low concentration and inactive or even activating at high concentration) was explained by hypothesizing (i) that quinones formed aggregates at high concentrations and (ii) that these aggregates were either inactive or activating compounds able to compete with the monomeric-inhibiting quinone.

The observation that the same concentration of ubiquinone analog can inhibit or activate PTP opening according to the cell line used no longer supports this model. These data are now more consistent with another proposed model [25] involving two regulatory sites: one responsible for inhibition and one for activation. The occupancy of a site by an active compound would, in turn, modulate PTP opening through secondary changes in the PTP Ca^2+^ binding affinity, whereas binding by an inactive compound would not. In this model, the biphasic response observed with some quinones could easily be explained through the assumption that these quinones may bind the two sites: the inhibitory site with high affinity and the inducing site with a lower affinity.

Because the effect of some ubiquinone analogs depends on the cell line, we propose that the affinity of these sites for one particular quinone, as well as the secondary changes in the PTP Ca^2+^ binding affinity, may change according to the cell line. These changes would depend on genetic or metabolic differences. Note, however, that the observed changes were not due to differences in the growth media (Figure 4), and that the CRC experiments were performed in the same experimental condition (Figure 1). Therefore, the putative modifications may directly affect the PTP.

This study constitutes the first report of a PTP-targeted strategy able to selectively open the PTP and consecutively provoke cell death in some cells whilst sparing others. Therefore, this work should be viewed as the first proof of concept suggesting that the PTP is a key target for selecting cells that will commit the cell death program. Finding compounds that open PTP in one cancerous cell line (without any side effect on normal cells) needs further studies. This will benefit from an improved knowledge of the molecular nature of the PTP, which in turn will deepen our understanding of why PTP-regulation by quinones (and probably other compounds) changes according to the cell line.
